# Teaching undergraduate students gynecological and obstetrical examination skills: the patient’s opinion

**DOI:** 10.1007/s00404-020-05615-1

**Published:** 2020-06-01

**Authors:** Amr Hamza, C. Warczok, G. Meyberg-Solomayer, Z. Takacs, I. Juhasz-Boess, E.-F. Solomayer, M. P. Radosa, C. G. Radosa, L. Stotz, S. Findeklee, J. C. Radosa

**Affiliations:** 1 Department of Obstetrics and Gynecology, Homburg University Medical Centre, 66421 Homburg, Germany; 2 Department of Radiology, Dresden University Hospital, Fetscherstraße 74, 01307 Dresden, Germany; 3 Department for Gynecology, Diaconia Clinic Kassel, Kassel, Hessen Germany

**Keywords:** Student, Education, Gynecology, Obstetrics

## Abstract

**Introduction:**

Our study assesses the patients’ opinion about gynecological examination performed by undergraduate students (UgSts). This assessment will be used in improving our undergraduate training program. A positive opinion would mean a lower chance of a patient refusing to be examined by a tutor or student, taking into account vaginal examination (VE).

**Materials and methods:**

We performed a prospective cross-sectional survey on 1194 patients, consisting of outpatient and inpatient at the departments of obstetrics and gynecology from November 2015 to May 2016. The questionnaire consisted of 46 questions. Besides demographic data, we assessed the mindset of patients regarding the involvement of undergraduate student (UgSt) in gynecological and obstetrical examinations. We used SPSS version 23 for the statistical analysis. For reporting the data, we followed the STROBE statement of reporting observational studies.

**Results:**

The median age was 38 years having a median of one child. 34% presented due to obstetrical problems, 38% due to gynecological complaints, and 19% due to known gynecological malignancies. Generally, we retrieved a positive opinion of patients towards the involvement of students in gynecological and obstetrical examination under supervision in 2/3 of the cases.

**Conclusions:**

There is no reason to exclude medical UgSts from gynecological and obstetrical examinations after obtaining a written or oral consent.

## Introduction

Undergraduate education has been a subject of an intense academic and social debate over the last few decades. “Foolish the doctor who despises the knowledge acquired by the ancients” was said by Hippocrates (c. 460—c. 380 B.C.), who shaped the cornerstones of today’s clinical management (history taking, physical examination and problem oriented management) [1]. Following his steps, Galen, Paracelsus, Sydenham, Boerhaave, Laennec, Graves, and countless other physicians developed the subset of clinical skills and the method of teaching them to the following generations. With their help, medical schools established the university curricula we know today [2]. The institutional development of the undergraduate educational system also underwent throughout history great changes due to various circumstances [3]. Yet, “the trend in many Universities has been for priority in promotion and recognition to be given excellence in research and scholarship rather than teaching” [4].

Clinical examination (CE) is the backbone of the daily medical practice. Teaching and learning CE has been shown to be safe in a well-surveyed setting [5]. Teaching clinical skills (CS) should, therefore, be the cornerstone of the undergraduate medical program [6–8].

Vaginal, abdominal, and breast examinations are basic gynecological examination skills, which students should be familiar with. Due to the intimacy of genital regions, teaching these CS is heavily debated. In contrast, in midwifery schools, gynecological examinations are uncontested and incorporated [9].

To facilitate its teaching, examination under anesthesia was suggested [10–14]. A prior written signed consent should be available [12, 15, 16]. Even though it is challenging to convey CS in the field of gynecology to medical students (MS), completely skipping it will downgrade the quality of undergraduate education [17].

We found three patient surveys, with low patient numbers, assessing student gynecological and mammary examinations with or without anesthesia. They revealed a general acceptance of student examination. No patient opinion surveys discussed gynecological examination conducted as part of an undergraduate teaching setting [18–21].

Therefore, most gynecologists defer from teaching undergraduates gynecological examinations [22, 23]. Not knowing whether patients might have agreed or not unnecessarily deprives the students of clinical skill training. Our survey was designed to fill the gap regarding patients’ opinion.

## Materials and methods

We designed a prospective cohort study of patients that were examined in our obstetrics and gynecology department between November 1st, 2015 and May 31st, 2016.

### Participants and settings

The University of Saarland Department of Obstetrics and Gynecology is a tertiary center in the state of Saarland, Germany. The outpatient clinic provides a high-end care, covering the whole spectrum of obstetrics and gynecology.

The survey is conducted on outpatients 18 years and older. We excluded patients that declined to participate, were unable to cooperate, or had language barriers. We also eliminated incomplete questionnaires that were less than 75% answered.

### Designing the questionnaire

The questionnaire surveys the patient’s opinion about undergraduate MS involvement in their examination. The questions surveyed all steps of this examination. Patients had to write their personal data and whether they had been victims of sexual abuse.

For the purpose of analysis, we divided teaching into passive and active teaching. Passive teaching is defined as the presence of the student while the physician examines a patient and explains the examination to the student allowing the student to ask questions. Active teaching allows the student to conduct the examination according to the physician’s instructions and supervision.

### The questionnaire

The questionnaire comprised 46 standardized questions. Patients were first asked if they principally support undergraduate education. The questionnaire was furthermore divided into two parts:

Part 1, surveying patient opinion about the acceptable extent of UgSt involvement in history taking, pelvic, abdominal and breast examination and US. For each examination form mentioned above, five standardized questions were asked. The questions were about the extent of involvement, i.e., starting with the mere presence, the possibility of explanation and asking questions during consultation, and the possibility of partly or fully overtaking the physician’s role with or without direct surveillance.

Part 2, surveying the patient characteristics. Patients’ demographics included the indication for referral or presentation, age, gravidity, marital status, country of origin, school degree, professional degree, and history of sexual harassment or rape.

### Statistical analysis

Using SPSS Version 23 (IBM Inc, Chicago, IL, USA), we used a Likert scale to standardize the patient’s answer, which was composed of four points: agree, likely to agree, likely to disagree, and disagree. After dichotomization, we analyzed the answer scales as categorical variables. To test the normality of distribution of the data, we used the Kolmogorov–Smirnov test. The data were not normally distributed. Therefore, we used the exact Fischer test for statistical analysis of the variables. A professional statistician conducted the statistical analysis.

## Ethical approval

The local ethical committee in the Medical Syndicate of Saarland, Saarbrücken (250/15) approved the methodology and ethicality of the study. The study was in accordance with the Declaration of Helsinki.

For reporting the data, we followed the STROBE statement of reporting observational studies [24].

## Results

The questionnaire was distributed to all patients presenting in the study period (*n* = 3056). A total of 1281 questionnaires were filled out and returned for further evaluation (response rate of 41.92%). 83 (6.48%) questionnaires were excluded due to incomplete data. Four questionnaires were omitted as they were filled by minors. Finally, 1194 (93.21%) questionnaires were available for statistical analysis.

**Fig. 1:**
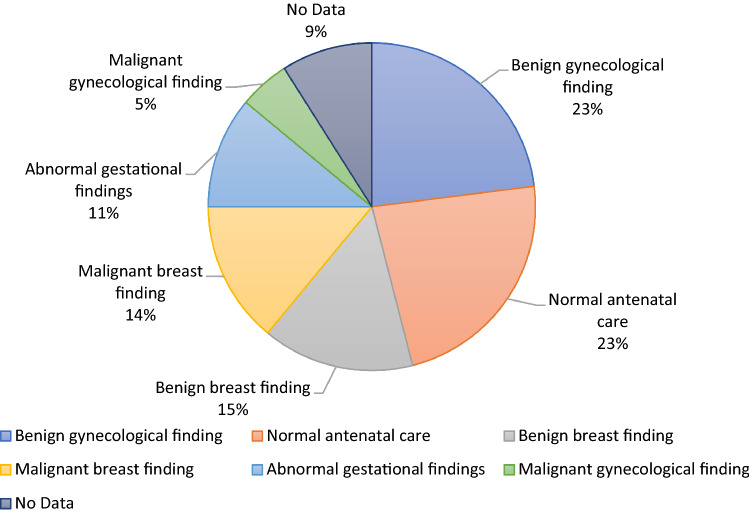
Pie chart presenting the indication for presentation in the outpatient clinic of the department of obstetrics and gynecology

The median age and parity of the patients were 38 years and one, respectively. The majority of surveyed patients were married or had a partner. Most of the patients were of German origin. Around half of the patients finished high school, the remaining patients graduated vocational school, first-phase secondary school and other comparable schooling. Almost half of the patients had higher professional degrees, while the other half had lower professional degrees. A minority had no professional degree. The majority of participants were not victims of sexual abuse or violence. An accurate patient demographics is presented in Table 1. The patients were presented to our tertiary center due to various reasons. The pie chart in Fig. 1 summarizes the various indications. The most common was benign gynecological finding, e.g., infertility or endometriosis followed by normal antenatal examinations. As presented in Fig. 1, pathological findings were less frequent.

**Table 1:** The demographic distribution of our patient collective that took part in the survey

	*n *= 1194 (%)
Social status	
Single	119 (10%)
Separated	61 (5%)
Married	712 (60%)
Has a partner	221 (18%)
Not declared	81 (7%)
Country of origin	
Germany	1013 (85%)
Other western European countries	18 (1.5%)
Eastern Europe	49 (4%)
Middle East	14 (1.2%)
Asia	22 (2%)
USA	0 (0%)
Latin America	3 (0.3%)
Not declared	75 (6%)
Educational level	
Did not finish school	13 (1%)
First-phase secondary school	202 (17%)
Vocational school	393 (33%)
High school	501(42%)
Other	12 (1%)
No data	73 (6%)
Professional degree	
None	95 (8%)
Professional experience	229 (19%)
1-Year master class	19 (2%)
2-Year master class	329 (27%)
University degree	262 (22%)
Other	128 (11%)
No input	132 (11%)

**Fig. 2:**
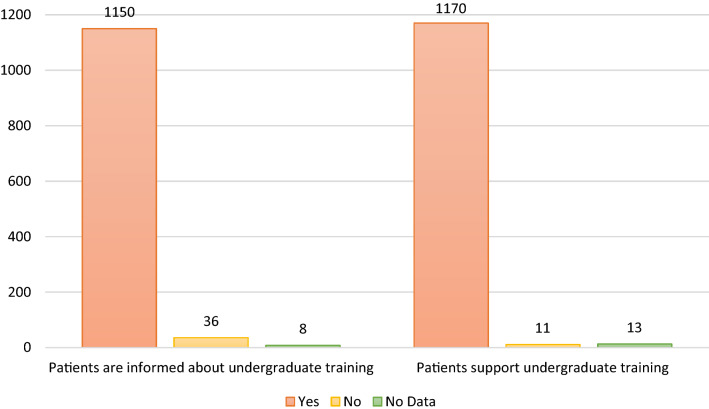
Column chart presenting that most of the patients knew about the presence of UgSts during their examination. Most of patients support undergraduate clinical training

**Fig. 3:**
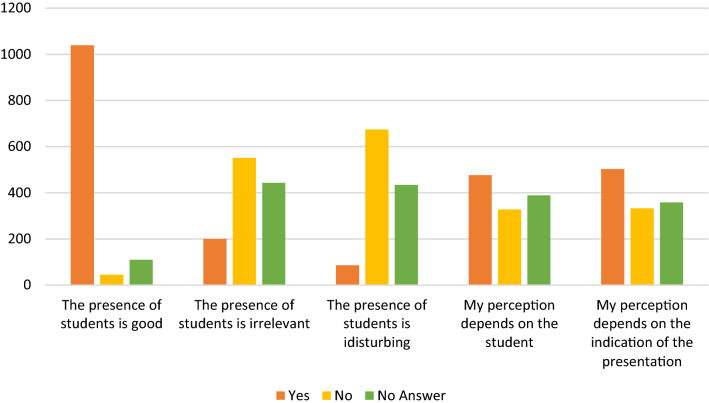
Column chart showing factors affecting their perception of students

1150 patients (96%) knew before arriving at the hospital that students may be present during their physical examination. 1170 (98%) were supporting undergraduate training as presented in Fig. 2. 1039 (87%) of the patients rated the presence of students as positive. A minority (86 patients; 7%) reported their presence as being disturbing. Between one-third and half of the patients thought that their perception depends on the student him/herself and the indication of their presentation in the clinic. These results are shown in Fig. 3.

Regarding history taking, most of the patients (983 patients; 82%) were in favor of students being present. 59% were in favor students actively taking the history, while 38% were not in favor of it. Regarding the general physical examination, e.g., blood pressure measurement, etc., 1070 patients (90%) were in favor of the presence of the students. 784 (66%) were in favor of students’ active participation during general medical examination. Surprisingly, 831 patients (70%) were in favor of the passive participation of students in the examination room during VE. More than expected agreed for the active participation by students (453 patients (38%)). During antenatal care, 960 patients (80%) and 756 (63%) supported the passive and active undergraduate training during transabdominal US, respectively. 994 (83%) and 692 (58%) patients supported the passive and active undergraduate training during breast examination, respectively. 834 patients (70%) supported the idea of being a patient for CE tests, e.g., OSCE. 461 (39%) even would allow VE as part of a medical test. 747 (63%) and 866 (73%) would agree to be part of breast examination or abdominal sonography medical exam, respectively. Most of the patients (783; 66%) think that this examination should be under surveillance. 358 patients (30%) even agree to have gynecological and obstetrical examination without surveillance.

Age played a significant role in the patient support for active teaching. Younger patients were statistically significantly more in favor of active teaching in history taking, general physical examination, VE and abdominal US than older patients. Regarding active teaching of breast examination and passive teaching in all fields there was no significant difference among different age groups. Parity did not seem to play a role on this matter, except for transabdominal sonography. Primigravidae tended to support more the active participation of students during transabdominal sonography. The marital status did not affect the patient’s opinion in almost all the examination and teaching forms. Yet, statistically significant more single patients agree for active participation in VE. The point of origin played a significant role in our study. European patients agreed statistically more to passive and active participation of students. Patients with higher educational level significantly agree more to active participation of general physical examination and abdominal US. Yet, patients with higher professional degree did not show any significant difference in their tolerance to being examined by students. To our surprise, being a victim of sexual harassment or violence did not affect patients on agreeing or disagreeing to active or passive participation of students in their medical examination. Based on the indication of their presentation, no significant difference was found among the different examinations. The underlying health status of patients did not play a significant role in deciding whether active or passive teaching can occur parallel to their examination. The relevant data are summed up in Table 2.

**Table 2:** The effect of age, health status, parity, marital status, point of origin, education, professional degree, and history of sexual harassment or violence on the incidence of supporting undergraduate involvement in CE

	Passive teaching		Active teaching	
	VE	BE	VE	BE
Health status				
< 30	27 (82%)	29 (91%)	16 (49%)	22 (69%)
31–70	253 (77%)	283 (87%)	126 (38%)	186 (57%)
> 70	457 (75%)	549 (90%)	248 (41%)	379 (62%)
* P* value	0.58	0.23	0.45	0.18
Age groups				
18–35	374 (75%)	434 (87%)	199 (40%)	298 (60%)
36–60	362 (78%)	415 (90%)	196 (42%)	295 (64%)
> 60	99 (72%)	125 (93%)	40 (29%)	78 (59%)
*P* value	0.26	0.15	0.02	0.37
Parity				
0	317 (75%)	368 (87%)	174 (41%)	255 (61%)
≥ 1	513 (77%)	600 (90%)	260 (39%)	413 (62%)
* P* value	0.51	0.24	0.53	0.66
Marital status				
No spouse	135 (77%)	156 (91%)	83 (47%)	105 (61%)
Has a spouse	675 (76%)	789 (89%)	343 (39%)	545 (61%)
*P* value	0.77	0.51	0.04	1.00
Point of origin patients				
Europe	793 (77%)	929 (90%)	422 (41%)	640 (62%)
Others	21 (57%)	25 (68%)	6 (16%)	15 (41%)
*P* value	0.01	0.01	< 0.01	0.01
Education				
Secondary school	145 (72%)	183 (89%)	70 (35%)	117 (58%)
Vocational school	296 (78%)	335 (90%)	149 (39%)	243 (65%)
High school	373 (76%)	434 (89%)	207 (42%)	295 (60%)
*P* value	0.30	0.90	0.22	0.17
Professional degree				
Patients only with work experience	342 (77%)	399 (90%)	171 (38%)	273 (62%)
Patients with technical master class	242 (77%)	290 (92%)	132 (42%)	208 (66%)
Patients with university degree	194 (76%)	219 (86%)	110 (43%)	147 (57%)
*P* value	0.99	0.06	0.45	0.11
History of sexual harassment or violence				
Yes	59 (75%)	71 (90%)	31 (39%)	49 (62%)
No	750 (76%)	874 (89%)	390 (40%)	603 (61%)
*P* value	0.78	1.00	1.00	1.00

## Discussion

The majority of patients are aware and support the undergraduate training during their examination. They agree to passive and active teaching of history taking, physical examination, mammary, transabdominal examination, and passive teaching of VE. More than one-third of the patients support active teaching of VE. They supported mostly the participation in undergraduate medical exams, e.g., objective structured CE (OSCE). Younger patients with higher educational level and European origin seem to approve more of undergraduate gynecological and obstetrical clinical skill teaching. The parity, marital status, history of sexual assault, or underlying health status did not affect the patient perception to active or passive teaching.

Medical examination is a cornerstone in undergraduate medical teaching. Yet the intimate nature of obstetrics and gynecology can be an obstacle to students learning this clinical skill. In our study, we were able to present the general perception and factors affecting patients’ opinion on agreeing to passive and active teaching.

The study took place in the biggest tertiary center in the district of Saarland. This gave us the chance to (a) cover the whole spectrum of management of obstetrics and gynecology and (b) generate a huge patient number. The multiplicity of treated diseases in the department provides our survey with the whole spectrum of Ob/Gyn patients.

The large study population renders our study more representative of the target population. To our knowledge, we recruited the largest number of participants in a survey covering these topics. Our sample size allowed us to perform a subgroup analysis [25], e.g., age and education. This may facilitate allocating teaching projects in the right patient groups and avoid embarrassing situations for students, patients, and their treating staff. We avoided performing a multiple subgroup analyses to avoid raising the likelihood of observing a false positive. This could lead to a flawed assumption that undergraduate teaching effect varies across subcategories [26].

The main problem with our study design is the confounding potential. As patients were not randomized to teaching groups, some patient characteristics were not evenly distributed between two groups. Non-European, unmarried, illiterate, and unemployed patients were underrepresented in our study. Despite the study population size and although we accounted for confounding variables, a certain degree of confounding might have occurred. Naturally, there is a potential for unmeasured confounding variables not known to the study group, as the questionnaire was standardized. Yet, due to the prospective nature of our questionnaire, we collected the most relevant and affecting data.

The questionnaire did not include non-German speakers, which posed another limitation to the study. Therefore, the study only reflected the behavior of German-speaking patients. Teaching a clinical skill requires a patient consent. The patient’s approval in turn requires a thorough understanding [13]. In a series of studies, certain cultural, religious, and other backgrounds may further hinder male students from examining female patients [27]. In our study, this subset of patients was underpowered; therefore, a conclusive statement is not possible.

38% of the patients agreed to be examined by an UgSt, which is significantly lower than other CS to be taught. In comparison to [28] 62% of the patients consented to be vaginally examined under anesthesia. This approach might be more successful in teaching VE to students. Another strategy is to incorporate simulators and phantoms in undergraduate studies. Yet, these devices are expensive and very complicated. Even if these teaching methods are close to reality, they are not entirely real and cannot replace a real-time CE [29, 30]. Gynecological teaching associates (GTAs) also seem to be effective in helping MS to learn VE. These are model patients that provide themselves for teaching purposes and were proven to be effective [22, 31].

Age, education level, and point of origin affected women’s willingness to allow active or passive teaching, while parity, civic status, history of sexual assault, or underlying health did not. Armitage et al. [6] stated recently that age, parity, or civic status did not affect the likelihood of a woman allowing a student to perform an intimate examination. Regarding women’s age, our studies showed divergent results. A possible explanation is the different population group and number of patients (1194 vs. 233). In their study, analysis by ethnicity was not performed due to missing data, which may also explain this confounding result. In Arnitage et al. study, they also observed the characteristics of the student. Being a female, older, relaxed in manner, smartly dressed, or engaging in history taking increased the likelihood of accepting a VE by a student. We did not analyze this factor. In other studies, increasing women’s age increased the likelihood of allowing pelvic examination [20, 32, 33]. In these studies, the number of participants was also less and the study population was different from ours.

In our study, less invasive examinations by students, e.g., physical examination or transabdominal sonography were more tolerated than invasive examination, e.g., VE. In another survey, student examination was less accepted in more invasive examinations in obstetrics and gynecology regarding, when compared to pediatrics and urology. Passaperuma et al. also pointed out that gender and the level of training of students affected the acceptance of their involvement [21]. In the survey of Hartz et al., most patients accepted student involvement. Even in their study, patients were more uncomfortable with a VE by student. Yet, with increasing visits, patients became more comfortable with the idea of being examined by students. The study group concluded that the patient–medical student relationship is a statistically significant parameter that affected their involvement [19].

## Conclusion

Our study shows that most of the surveyed outpatients are willing to passively and actively involve students in their examination. In case of VE, less patients agreed to active teaching. A higher educational degree, young age, and European origin are parameters that increase the likelihood for agreeing to active and passive teaching. Parity, marital status, history of sexual assault, or underlying health status did not affect the patient’s perception of active or passive teaching. Based on other studies, VE under anesthesia after consenting and a good student–patient relationship may further improve the students’ clinical training. Furthermore, there is no reason to exclude medical UgSts from gynecological and obstetrical examinations after obtaining a written or oral consent.

## Abbreviations

CE: Clinical examination
CS: Clinical skills
MS: Medical students
UgSt: Undergraduate student
US: Ultrasound
VE: Vaginal examination

## References

[1] Gleeson F (1993). Clinical teaching: past–present–future. Ir J Med Sci.

[2] Bloch H (1995). Hermann Boerhaave (1668–1738): Life, teacher, practitioner, and activator of hippocratic clinical teaching. Heart Lung.

[3] Conway H (2002). The Medical Faculty, University of Glasgow Evolution of Clinical Teaching in the Late 19th and Early 20th Centuries. Scott Med J.

[4] Meador KJ (2015). Decline of clinical research in academic medical centers. Neurology.

[5] Mol SSL, Peelen JH, Kuyvenhoven MM (2011). Patients’ views on student participation in general practice consultations: a comprehensive review. Med Teach.

[6] Armitage AJ, Cahill DJ (2018). Medical students and intimate examinations: What affects whether a woman will consent?. Med Teach.

[7] Hamza A, Solomayer EF, Takacs Z, Juhasz-Boes I, Joukhadar R, Radosa JC (2016). Introduction of basic obstetrical ultrasound screening in undergraduate medical education. Arch Gynecol Obstet.

[8] Mavrova R, Radosa JC, Wagenpfeil G, Hamza A, Solomayer EF, Juhasz-Boss I (2016). Learning curves for laparoscopic hysterectomy after implementation of minimally invasive surgery. Int J Gynaecol Obstet: Off Organ Int Fed Gynaecol Obstet.

[9] Fullerton JT, Ghérissi A, Johnson PG, Thompson JB (2011). Competence and competency: core concepts for international midwifery practice. Int J Childbirth.

[10] Goedken J (2005). Pelvic examinations under anesthesia: an important teaching tool. J Health Care Law Policy.

[11] Wilson RF (2005). Autonomy suspended: using female patients to teach intimate exams without their knowledge or consent. J Health Care Law Policy.

[12] Wall LL, Brown D (2004). Ethical issues arising from the performance of pelvic examinations by medical students on anesthetized patients. Am J Obstet Gynecol.

[13] Ubel PA, Jepson C, Silver-Isenstadt A (2003). Don't ask, don't tell: a change in medical student attitudes after obstetrics/gynecology clerkships toward seeking consent for pelvic examinations on an anesthetized patient. Am J Obstet Gynecol.

[14] Bibby J, Boyd N, Redman CW, Luesley DM (1988). Consent for vaginal examination by students on anaesthetised patients. Lancet.

[15] Wilson RF (2005). Autonomy suspended: using female patients to teach intimate exams without their knowledge or consent. J Health Care L Pol'y.

[16] Goedken J (2005). Pelvic examinations under anesthesia: an important teaching tool. J Health Care L Pol'y.

[17] Lamb D (2007). Could simulated emergency procedures practised in a static environment improve the clinical performance of a Critical Care Air Support Team (CCAST)?: a literature review. Intensive Crit Care Nurs.

[18] Thurman AR, Litts PL, O'Rourke K, Swift S (2006). Patient acceptance of medical student participation in an outpatient obstetric/gynecologic clinic. J Reprod Med.

[19] Hartz MB, Beal JR (2000). Patients’ Attitudes and Comfort Levels Regarding Medical Students’ Involvement in Obstetrics—Gynecology Outpatient Clinics. Acad Med.

[20] Koehler N, McMenamin C (2012). Would you consent to being examined by a medical student? Western Australian general public survey. Med Teach.

[21] Passaperuma K, Higgins J, Power S, Taylor T (2008). Do patients’ comfort levels and attitudes regarding medical student involvement vary across specialties?. Med Teach.

[22] Dugoff L, Pradhan A, Casey P, Dalrymple JL, Abbott JF, Buery-Joyner SD (2016). Pelvic and breast examination skills curricula in United States medical schools: a survey of obstetrics and gynecology clerkship directors. BMC Med Educ.

[23] Seago BL, Ketchum JM, Willett RM (2012). Pelvic examination skills training with genital teaching associates and a pelvic simulator: does sequence matter?. Simul Healthc : J Soc Simul Healthc.

[24] von Elm E, Altman DG, Egger M, Pocock SJ, Gotzsche PC, Vandenbroucke JP (2008). The Strengthening the Reporting of Observational Studies in Epidemiology (STROBE) statement: guidelines for reporting observational studies. J Clin Epidemiol.

[25] Pocock SJ, Assmann SE, Enos LE, Kasten LE (2002). Subgroup analysis, covariate adjustment and baseline comparisons in clinical trial reporting: current practice and problems. Stat Med.

[26] Lagakos SW (2006). The challenge of subgroup analyses–reporting without distorting. N Engl J Med.

[27] Alam K, Safdar CA, Munir TA, Ghani Z (2014). Teaching obstetrics and gynaecology to male undergraduate medical students: student's perception. J Ayub Med Coll Abbottabad.

[28] Wainberg S, Wrigley H, Fair J, Ross S (2010). Teaching pelvic examinations under anaesthesia: what do women think?. J Obstet Gynaecol Can.

[29] Seago BL, Ketchum JM, Willett RM (2012). Pelvic examination skills training with genital teaching associates and a pelvic simulator: does sequence matter?. Simul Healthc.

[30] Dilaveri C, Szostek J, Wang A, Cook DA (2013). Simulation training for breast and pelvic physical examination: a systematic review and meta-analysis. BJOG: Int J Obstet Gynaecol.

[31] Janjua A, Smith P, Clark TJ (2018). A cross-sectional study on teaching pelvic examination in medical schools in the UK (the COTES study). J Obstet Gynaecol.

[32] Racz JM, Srikanthan A, Hahn PM, Reid RL (2008). Gender preference for a female physician diminishes as women have increased experience with intimate examinations. J Obstet Gynecol Can: JOGC.

[33] Mills JK, Lambert KV, Krupa J (2015). Medical students in breast clinics–how welcome are they and how can we improve their learning opportunities?. J Surg Educ.

