# Correction to: Teaching undergraduate students gynecological and obstetrical examination skills: the patient’s opinion

**DOI:** 10.1007/s00404-021-06324-z

**Published:** 2021-11-16

**Authors:** Amr Hamza, C. Warczok, G. Meyberg-Solomayer, Z. Takacs, I. Juhasz-Boess, E.-F. Solomayer, M. P. Radosa, C. G. Radosa, L. Stotz, S. Findeklee, J. C. Radosa

**Affiliations:** 1 Department of Obstetrics and Gynecology, Homburg University Medical Centre, 66421 Homburg, Germany; 2 Department of Radiology, Dresden University Hospital, Fetscherstraße 74, 01307 Dresden, Germany; 3 Department for Gynecology, Diaconia Clinic Kassel, Kassel, Hessen Germany

## Correction to: Archives of Gynecology and Obstetrics (2020) 302:431–438 10.1007/s00404-020-05615-1

The article “Teaching undergraduate students gynecological and obstetrical examination skills: the patient’s opinion” written by Amr Hamza, C. Warczok, G. Meyberg-Solomayer, Z. Takacs, I. Juhasz-Boess, E.-F. Solomayer, M. P. Radosa, C. G. Radosa, L. Stotz, S. Findeklee and J. C. Radosa was originally published electronically on the publisher’s internet portal on June 1, 2020 without open access. With the author(s)’ decision to opt for Open Choice the copyright of the article changed to © The Author(s) 2020 and the article is forthwith distributed under a Creative Commons Attribution 4.0 International License, which permits use, sharing, adaptation, distribution and reproduction in any medium or format, as long as you give appropriate credit to the original author(s) and the source, provide a link to the Creative Commons licence, and indicate if changes were made. The images or other third-party material in this article are included in the article’s Creative Commons licence, unless indicated otherwise in a credit line to the material. If material is not included in the article’s Creative Commons licence and your intended use is not permitted by statutory regulation or exceeds the permitted use, you will need to obtain permission directly from the copyright holder. To view a copy of this licence, visit http://creativecommons.org/licenses/by/4.0/. Open Access funding enabled and organized by Projekt DEAL.

The original article has been updated.

